# Higher Initial DNA Damage and Persistent Cell Cycle Arrest after Carbon Ion Irradiation Compared to X-irradiation in Prostate and Colon Cancer Cells

**DOI:** 10.3389/fonc.2016.00087

**Published:** 2016-04-13

**Authors:** Annelies Suetens, Katrien Konings, Marjan Moreels, Roel Quintens, Mieke Verslegers, Els Soors, Kevin Tabury, Vincent Grégoire, Sarah Baatout

**Affiliations:** ^1^Expert Group for Molecular and Cellular Biology, Radiobiology Unit, Belgian Nuclear Research Centre (SCK•CEN), Institute for Environment, Health and Safety, Mol, Belgium; ^2^Radiation Oncology Department, Center for Molecular Imaging, Radiotherapy and Oncology, Institut de Recherche Expérimentale et Clinique (IREC), Université Catholique de Louvain (UCL), Bruxelles, Belgium; ^3^Laboratory of Experimental Radiotherapy, Department of Oncology, KU Leuven, Leuven, Belgium

**Keywords:** carbon ion irradiation, PC3, Caco-2, cell cycle progression, DNA double-strand break damage and repair

## Abstract

The use of charged-particle beams, such as carbon ions, is becoming a more and more attractive treatment option for cancer therapy. Given the precise absorbed dose-localization and an increased biological effectiveness, this form of therapy is much more advantageous compared to conventional radiotherapy, and is currently being used for treatment of specific cancer types. The high ballistic accuracy of particle beams deposits the maximal dose to the tumor, while damage to the surrounding healthy tissue is limited. In order to better understand the underlying mechanisms responsible for the increased biological effectiveness, we investigated the DNA damage and repair kinetics and cell cycle progression in two p53 mutant cell lines, more specifically a prostate (PC3) and colon (Caco-2) cancer cell line, after exposure to different radiation qualities. Cells were irradiated with various absorbed doses (0, 0.5, and 2 Gy) of accelerated ^13^C-ions at the Grand Accélérateur National d’Ions Lourds facility (Caen, France) or with X-rays (0, 0.1, 0.5, 1, 2, and 5 Gy). Microscopic analysis of DNA double-strand breaks showed dose-dependent increases in γ-H2AX foci numbers and foci occupancy after exposure to both types of irradiation, in both cell lines. However, 24 h after exposure, residual damage was more pronounced after lower doses of carbon ion irradiation compared to X-irradiation. Flow cytometric analysis showed that carbon ion irradiation induced a permanent G2/M arrest in PC3 cells at lower doses (2 Gy) compared to X-rays (5 Gy), while in Caco-2 cells the G2/M arrest was transient after irradiation with X-rays (2 and 5 Gy) but persistent after exposure to carbon ions (2 Gy).

## Introduction

Over the past decades, an increase in the use of hadrontherapy has been observed (1). Hadrontherapy uses accelerated particles, such as protons or carbon ions, thereby offering a ballistic advantage during treatment. The inverted depth–dose profile and a sharp dose fall-off result in a precise dose-localization called Bragg peak (2). As such, a very specific energy deposition is focused on the tumor, while the surrounding healthy tissue is spared to a maximum. When carbon ions are used, the high-linear energy transfer (LET) also offers biological advantages compared to X-irradiation (3). From a physical point of view, low-LET photon irradiation deposits its energy in a disperse manner. This homogeneous distribution of energy in the irradiation field strongly relies on secondary ionizations (by the formation of reactive oxygen species) in the cell that will indirectly induce DNA damage homogeneously. By contrast, with particle irradiation, energy is not released in a disperse manner but rather along the track of the beam. Therefore, damage is more straightforward along the track that induces more complex and clustered DNA damage via a direct mechanism (4, 5). In view of therapeutic measures, the induction of DNA damage and specifically the double-strand break (DSB) is seen as the most prominent target in order to destroy cancer cells (6). Since DNA damage induced by high-LET radiation is more complex compared to low-LET irradiation, the relative biological effectiveness (RBE) of particle beams will be higher compared to X-rays (6). In this regard, it has been shown that hadrontherapy with carbon ions is more cytotoxic due to the higher RBE compared to photon irradiation (7, 8). However, the specific impact of carbon ion irradiation on cell cycle changes and comparison with X-irradiation in PC3 and Caco-2 cancer cells has not been investigated so far.

When DNA damage is induced, DSBs are detected in the cell by sensing molecules, such as DNA-dependent protein kinases (DNA-PK) or Ku70, which activate a signaling cascade by phosphorylating the histone H2AX (γ-H2AX) (9, 10). Another sensing molecule that is activated after DNA damage is p53, also known as the guardian of the genome (11). Repair enzymes will be attracted to the damaged site and the cell will go into cell cycle arrest to allow time for repair. It is well known that the number of γ-H2AX foci is proportional to the amount of DSBs (12–14). By immunofluorescent staining of the γ-H2AX foci, quantitative and qualitative evaluation of the damage can be performed. A previous *in vitro* study investigating the differential effect of high- and low-LET radiation has shown that the initial formation (as early as 15 min) of γ-H2AX foci is similar for equal doses of different beam qualities (15). However, repair kinetics (investigated at later time points) have shown a delayed or less successful repair of DSBs after high-LET radiation (16, 17). Therefore, particle irradiation can be effective in inducing cell death even in highly radioresistant cells (18). One of the factors that plays a major role in determining radiosensitivity is p53. Mutations or deletions in the p53 gene can lead to the radioresistance of cancer cells to conventional radiotherapy (19–22). By contrast, previous studies with high-LET radiation have shown that this type of radiation can induce apoptosis effectively regardless of p53 gene status (7, 23).

*In vitro* studies comparing the effect of particle or photon irradiation have shown a more pronounced cell cycle arrest induced by particles (24, 25). Furthermore, it has been shown that cells are more sensitive to the induction of DSBs by X-irradiation during the G2/M-phase of the cell cycle (26). Contrarily, the radiation sensitivity of cancer cells irradiated with particles is less, but not entirely, dependent on the cell cycle stage (27). Thus, particle beam therapy is more suitable to damage a heterogeneous tumor population, consisting of cells in different cell cycle stages (24).

We previously investigated the transcriptional response of PC3 and Caco-2 cells after X- and carbon ion irradiation, in which we observed more pronounced changes in gene expression after carbon ion irradiation. Genome-wide analysis in PC3 cells showed that gene sets involved in cell cycle regulation and, interestingly, also in motility processes were found to be modulated, especially after carbon ion irradiation (28). In a next step, we further investigated the changes of genes involved in motility processes. Our results showed that the magnitude of expression of these genes was time- and dose-dependent for both PC3 and Caco-2 cells, although a cell-type-specific response to X- and carbon ion irradiation was observed (29). With regard to the changes in cell cycle-related gene sets, we further aimed to investigate the acute cellular responses induced by different radiation qualities. Therefore, in this study, we examined both DNA repair kinetics and cell cycle progression in PC3 and Caco-2 cells in response to carbon ion or X-irradiation. Cells were irradiated with different doses ranging from 0.1 up to 5 Gy depending on the type of radiation. DNA damage and repair kinetics were analyzed up to 24 h after irradiation and cell cycle progression up to 72 h after irradiation. Further elucidation of the effect of different beam qualities on different cancer cell lines will contribute to a better understanding of which therapy would be most suited for these types of cancers.

## Materials and Methods

### Cell Culture

Human prostate adenocarcinoma cells (PC3; ATCC^®^ CRL-1435™) and colorectal adenocarcinoma cells (Caco-2; ATCC^®^ HTB-37™) were obtained from the American Type Culture Collection (ATCC, Molsheim Cedex, France). PC3 cells were cultured in Kaighn’s Modification of Ham’s F-12 Medium (F-12K) (ATCC) supplemented with 10% fetal bovine serum (FBS) (GIBCO, Life Technologies, Ghent, Belgium), as specifically recommended by ATCC. Caco-2 cells were cultured in Dulbecco’s Modified Eagle medium (DMEM) (GIBCO) supplemented with 10% FBS and 1% non-essential amino acids (GIBCO). Cell cultures were maintained in a humidified incubator (37°C; 5% CO_2_). For all irradiation experiments, the same passage number of cells was used. Cell doubling time was 26 and 20 h for PC3 and Caco-2 cells, respectively (data not shown). Cell cultures were regularly tested for mycoplasma contamination (DSMZ, Braunschweig, Germany).

### X-irradiation

X-irradiation experiments were performed at the irradiation facility available at SCK•CEN (Mol, Belgium). Medium was replaced prior to irradiation in a horizontal position. Cells were exposed to different doses of X-rays (0, 0.1, 0.5, 1, 2, and 5 Gy) using a Pantak HF420 RX machine (250 kV, 15 mA, 1.2 mm Aluminum equivalent, 1 mm Cu-filtered X-rays, and a calculated dose rate of 0.25 Gy/min). The beam quality of H-250 (as recommended by ISO 4037-1) was used. This beam quality was created using a tube voltage of 250 kV and 1 mm Cu additional filtration. The secondary standard for X-rays is the NE2571 0.6 cc ionization chamber SN309 connected to Keithley 6517B SN1335646 electrometer. The calibration of this chamber in terms of air Kerma (K_air_), for H-250 beam quality, was done in 2013 at the primary standard laboratory PTB, Germany. The reference quantity is K_air_ in one point, taken as the reference position of the irradiated sample, which typically is its center. No correction is done for the extended volume and self-absorption of the sample itself and such effect is not included in the uncertainties budget either. The irradiation is based on the ISO 4037 standard. All uncertainties are the expanded uncertainties for *k* = 2 (confidence level 95%). The dose rate was measured for each distance, by using repeatedly the same distance, one relies on stability from 1 day to another and, therefore, only periodic checks of beam stability are performed at the irradiation facility.

### Carbon Ion Irradiation

For our experiment, we were assigned ^13^C beam time at the Grand Accélérateur National d’Ions Lourds (GANIL) (Caen, France). Cells were transported by car in a transportable incubator at 37°C to GANIL. For all assays, 10^5^ cells were plated in 12.5 cm^2^-tissue culture flasks (Falcon; VWR; Leuven, Belgium) 3 days before transport, during which all culture flasks were completely filled with medium. After arrival, medium was changed, and cells were placed overnight in a humidified incubator. Before the irradiation, culture flasks were completely filled with medium to allow irradiation in a vertical position, perpendicular to a horizontal carbon ion beam. The cells were irradiated with a ^13^C beam with an initial energy of 75 MeV/u (LET = 33.7 keV/μm). The applied doses were 0, 0.5, 1, and 2 Gy. Carbon ion dosimetry was performed as previously described (28, 30). The RBE of carbon ions at 10% survival was 1.67 for PC3 cells and 1.83 for Caco-2 cells (29).

### Immunocytochemistry for γ-H2AX

For X-irradiation experiments, cells were plated on coverslips at a density of 20,000 cells/well and grown for 2 days. Due to practical reasons, samples were irradiated in T12.5 flasks for the carbon ion irradiation (vertical position). Irradiation with both radiation qualities was then performed with a series of doses as mentioned before. At various time points after irradiation (30 min, 1, 2, 4, 8, and 24 h), cells were fixed in 4% paraformaldehyde (Merck KGaA, Darmstadt, Germany) for at least 20 min at 4°C. Afterwards, cells were washed with PBS and permeabilized in 0.25% Triton (Sigma-Aldrich Co.) in PBS for 3 min. Subsequently, cells were probed with mouse anti-γ-H2AX antibody (ab26350, Abcam, Cambridge, UK) (1:300 dilution) and incubated overnight at 4°C. Next, the cells were washed with PBS and stained with Alexa Fluor 488 goat anti-mouse (H + L)-labeled antibody (A11001, Invitrogen, Life technologies) (1:300 dilution) for 2 h at room temperature. All antibody dilutions were prepared in 3% bovine serum albumin (BSA). Following this, three washing steps were performed with PBS after which a cover glass was mounted on the samples with Vectashield containing 4′,6-diamidino-2-phenylindole (DAPI) (Vector Laboratories, Brussels, Belgium).

### Automated Fluorescence Microscopy and Image Analysis

Images were acquired with a Nikon Eclipse Ti (automated inverted wide-field epifluorescence microscope) equipped with a 40× magnification (Plan Fluor, numerical aperture 1.3) oil objective and a Nikon TE2000-E camera controlled by the NIS Elements software. The images were taken in the same orientation as the irradiation was performed, i.e., the viewer position was perpendicular to the cellular plane. Per condition a mosaic of 25 fields was acquired with a lateral spacing of 190 μm between fields (corresponding to the size of the field of view) and each field was acquired as a z-stack of nine planes axially separated by 1 μm. Images were analyzed with Fiji software (31) using the InSCyDe-02 toolbox. The software allowed to analyze each nucleus based on the DAPI signal. Within each nucleus, pixel size and intensity emitted from the Alexa 488 fluorochrome were analyzed after which the γ-H2AX foci number per nucleus and the foci occupancy are determined in a fully automatic manner. These data were then used to count the radiation-induced damage, i.e., subtract the damage of control cells from irradiated cells. As mentioned before, for carbon ion irradiation experiments, cells were seeded in T12.5 flasks (plastic surface) since these samples were irradiated in a vertical position. X-irradiated samples were seeded on glass cover slips for γ-H2AX. As a result, image quality was less good for carbon ion samples, and as a consequence Fiji software was unable to correctly count the number of spots in each nucleus for the carbon ion-irradiated samples. Therefore, we decided to count the spots manually for the carbon ion samples. At least 170 and 100 nuclei were analyzed per sample for X-ray and carbon ion irradiation, respectively.

### Cell Cycle Analysis

**Figure 1:**
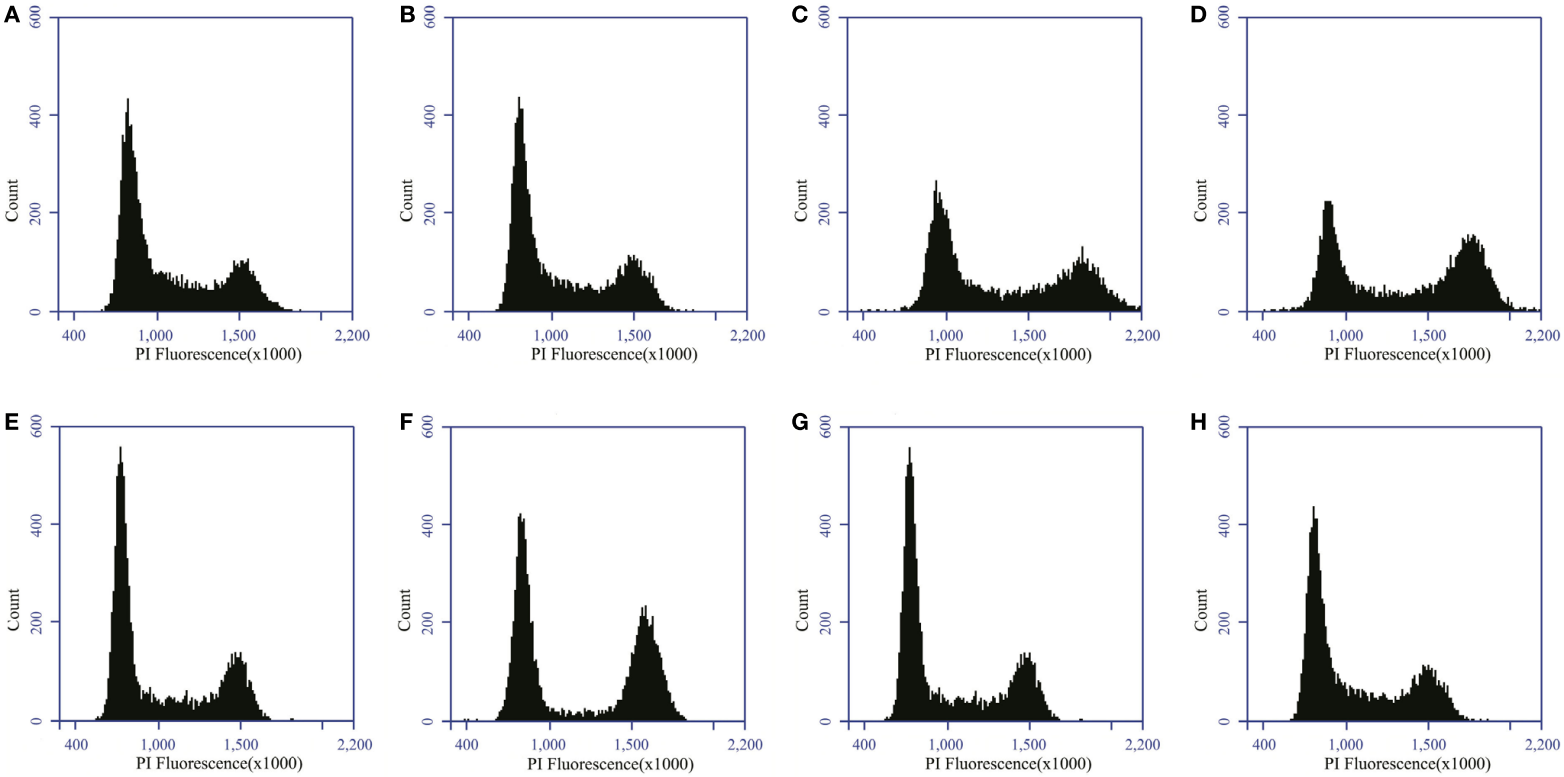
**Distribution of PC3 and Caco-2 cells in the different phases of the cell cycle**. Distribution of PC3 cells in the cell cycle for control **(A)** and 2 Gy X-ray irradiated **(B)** samples 24 h after irradiation. Distribution of Caco-2 cells in the cell cycle for control **(C)** and 2 Gy X-ray-irradiated **(D)** samples 24 h after irradiation. Distribution of PC3 cells in the cell cycle for control **(E)** and 2 Gy carbon ion-irradiated **(F)** samples 24 h after irradiation. Distribution of Caco-2 cells in the cell cycle for control **(G)** and 2 Gy carbon ion-irradiated **(H)** samples 24 h after irradiation.

Cells were collected at 24, 48, and 72 h after irradiation by use of trypsinization. In addition, supernatants and PBS used during wash steps were kept as well to ensure the collection of both adherent and detached cells. After collection, samples were fixed in a cold 80% EtOH solution at 4°C for at least 1 h. Fixed samples obtained in GANIL were transported back to SCK•CEN for further processing. Next, samples were washed with PBS and stained with a 500 μl propidium iodide (PI) solution (50 μg/ml PI + 1% RNase A) (Sigma-Aldrich Co. LLC; Bornem; Belgium) for 50 min at 37°C. Samples were measured immediately afterwards by flow cytometry (Accuri C6 system; BD Biosciences, Erembodegem, Belgium). PI fluorescence of a minimum of 10,000 cells was measured. Cells in G0/G1, S, and G2/M-phase were determined after filtering for doublets and aggregates. Doublets were filtered based on a FSC-A vs. FSC-H dot plot with Accuri C6 software. In addition, sub G1 cells were identified as cells with a DNA content of between half the mean value of G1 phase and the minimum value of G1 phase. Based on the histogram, we determined the peak of G1, on which the settings were placed in such a way that 90% falls within the peak. The peak of G2 needs to be 2 × G1 and also for this the settings were placed in such a way that 90% falls within the peak. Everything in-between was seen as S-phase. Everything in-between 0.5 × G1 and the beginning of G1 phase was the sub G1 peak. Re-analysis of samples was performed with ModFit LT software (Verity Software House, Topsham, ME, USA). Representative histograms are visualized in Figure 1.

### Statistical Analysis

Cell cycle data were analyzed by two-way analysis of variance (ANOVA) with dose and time point as independent variables. Analysis of γ-H2AX foci count data was performed using Kruskal–Wallis and *post hoc* Dunn’s multiple comparison tests. All analyses were performed using GraphPad Prism 5.00 software. For all tests, a value of *p* < 0.05 was considered statistically significant.

## Results

### DNA Damage and Repair Kinetics

**Figure 2:**
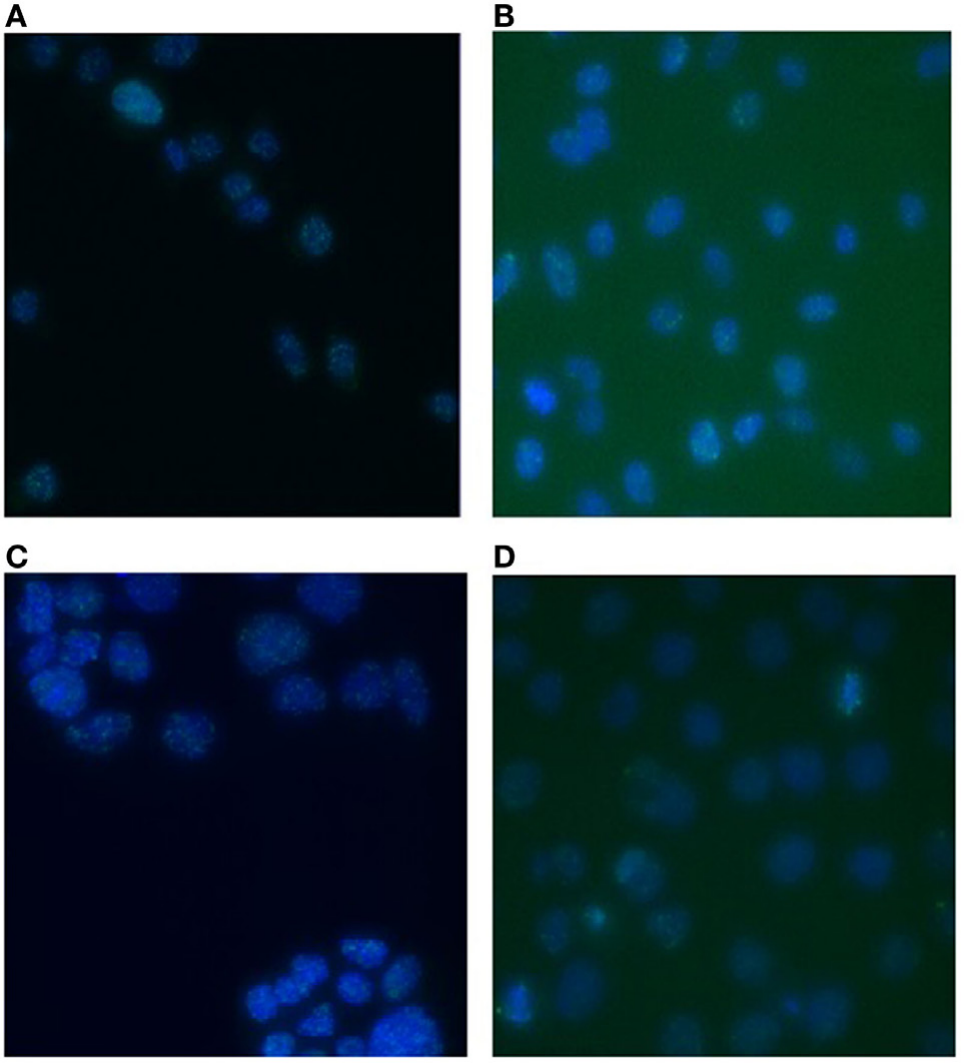
**γ-H2AX foci in PC3 and Caco-2 cells after irradiation with X-rays or carbon ions**. Representative images of γ-H2AX foci in PC3 cells 1 h after 2 Gy X-irradiation **(A)** and 1 h after 2 Gy carbon ion irradiation **(B)**. Representative images of γ-H2AX foci in Caco-2 cells 1 h after 2 Gy X-irradiation **(C)** and 1 h after 2 Gy carbon ion irradiation **(D)**. Images were acquired with a Nikon Eclipse Ti (automated inverted wide-field epifluorescence microscope) equipped with a 40× magnification (Plan Fluor, numerical aperture 1.3) oil objective and a Nikon TE2000-E camera controlled by the NIS Elements software.

**Figure 3:**
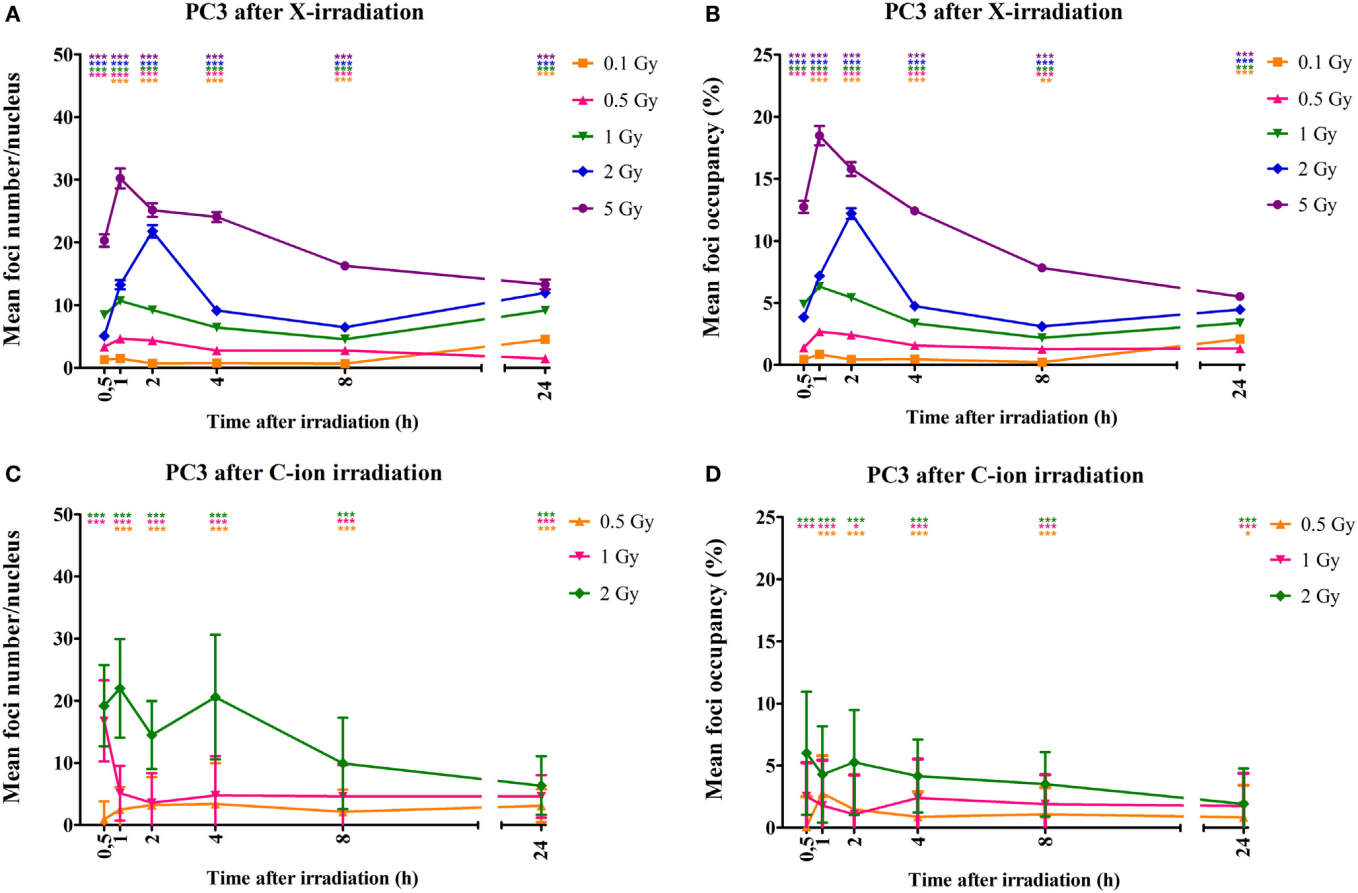
**Quantification of γ-H2AX foci number and occupancy in X- and carbon ion-irradiated PC3 cells**. Dots representing mean γ-H2AX foci number per nucleus vs. time **(A)** and mean foci occupancy per nucleus vs. time **(B)** after X-irradiation in PC3 cells. Dots representing mean γ-H2AX foci number per nucleus vs. time **(C)** and mean foci occupancy per nucleus vs. time **(D)** after exposure to carbon ions. Fiji software was used to count the number of nuclei and foci occupancy in each nucleus. The number of foci in non-irradiated cells was subtracted from that of irradiated cells for each dose and time point. For X-rays, the error bars represent the SEM of three independent experiments; for carbon ion data, the error bars represent STDEV of the experiment. Statistical Kruskal–Wallis analysis with Dunn’s multiple comparison tests were performed in GraphPad with *p < 0.05 (vs. control cells), **p < 0.01 (vs. control cells), and ***p < 0.001 (vs. control cells).

DNA DSBs were visualized by immunofluorescent staining for γ-H2AX foci that were analyzed at various time points (30 min, 1, 2, 4, 8, and 24 h) after irradiation. Representative images of the γ-H2AX foci for both PC3 and Caco-2 are shown in Figure 2. We counted both the number of radiation-induced foci, as a measure of DSBs, and the foci occupancy because H2AX phosphorylation as well as the size of foci differs throughout the cell cycle (32). Upon irradiation, a clear dose-dependent induction in the number and nuclear occupancy of foci was observed. A significant dose-dependent increase in foci number was detected after X-irradiation in PC3 cells as early as 30 min after irradiation (Figure 3A). Increased foci numbers induced by irradiation were associated with a higher percentage of the area of the nucleus ­covered by foci as seen in the elevated foci occupancy (Figure 3B). A follow-up of foci number and foci occupancy over time ­evidenced ­time-dependent repair of foci (Figures 3A,B). Maximum foci numbers were detected 1 h after X-irradiation (Figure 3A), after which repair seems to have initiated. Interestingly, most γ-H2AX foci were repaired 24 h after X-irradiation with doses up to 0.5 Gy, while residual foci were still visible after exposure to higher X-ray doses (Figures 3A,B). For carbon ion irradiation, the number of foci was still significantly elevated at 24 h after irradiation after all doses in PC3 cells (Figure 3C). Maximum foci numbers were detected 1 h after irradiation with carbon ions. A similar trend was observed for the foci occupancy in PC3 cells (Figure 3D).

**Figure 4:**
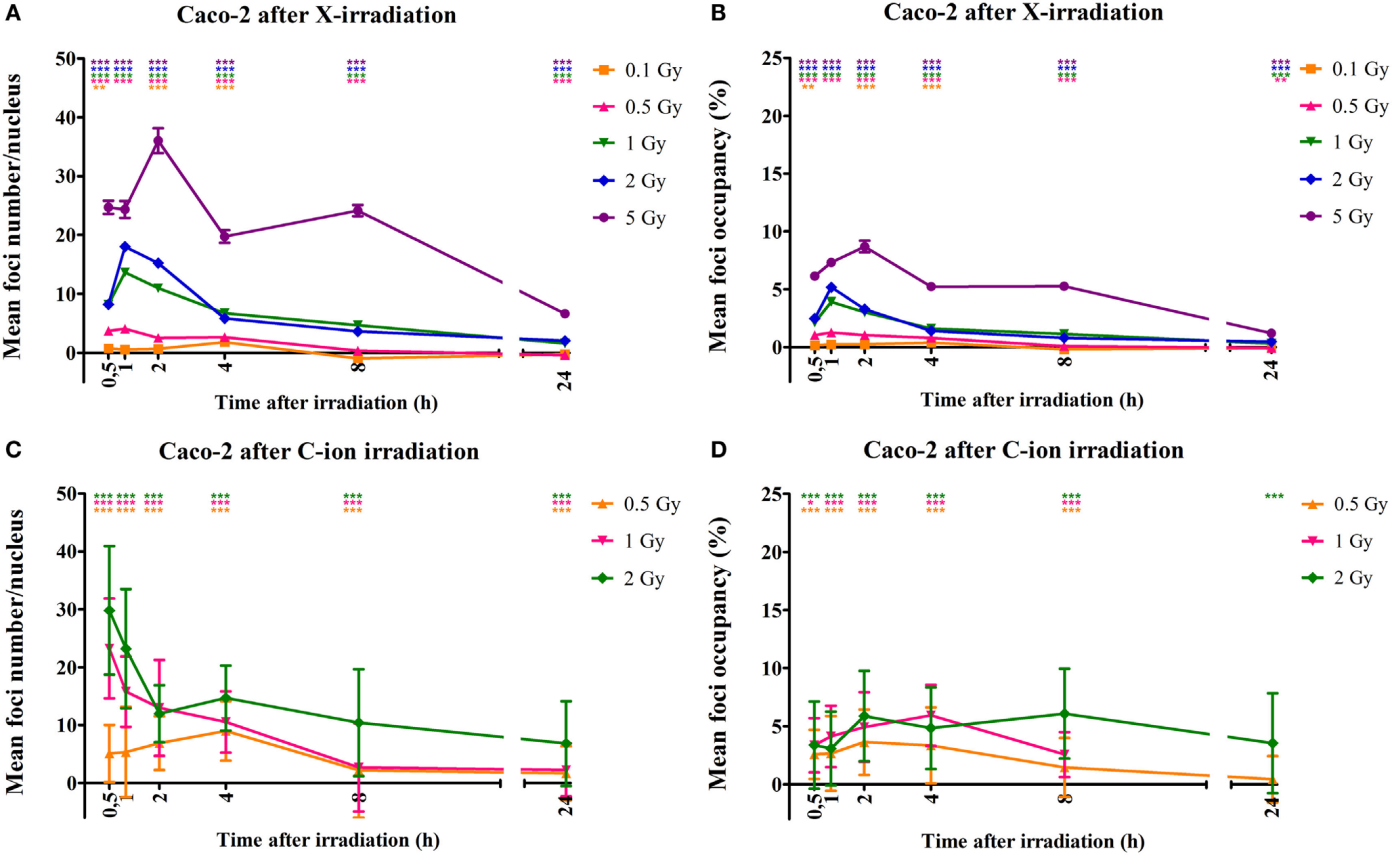
**Quantification of γ-H2AX foci number and occupancy in X- and carbon ion-irradiated Caco-2 cells**. Dots representing mean γ-H2AX foci number per nucleus vs. time **(A)** and mean foci occupancy per nucleus vs. time **(B)** after X-irradiation in Caco-2 cells. Dots representing mean γ-H2AX foci number per nucleus vs. time **(C)** and mean foci occupancy per nucleus vs. time **(D)** after exposure to carbon ions. Fiji software was used to count the number of nuclei and foci occupancy in each nucleus. For X-rays, the error bars represent the SEM of three independent experiments; for carbon ion data, the error bars represent STDEV of the experiment. Statistical Kruskal–Wallis analysis with Dunn’s multiple comparison tests were performed in GraphPad with **p < 0.01 (vs. control cells), ***p < 0.001 (vs. control cells).

Similar results were observed for the Caco-2 cells. More specifically, a dose-dependent increase in foci number was observed as early as 30 min after X-irradiation (Figure 4A). This increase was accompanied by an increase in foci occupancy (Figure 4B). Maximum foci numbers were observed at 1 to 2 h after X-irradiation after which a time-dependent repair was evidenced (Figure 4A). For the Caco-2 cells, 24 h after X-irradiation residual foci were still present for doses up to 1 Gy (Figure 4A). Similar observations were made for carbon ion-irradiated Caco-2 cells, where significantly elevated foci number were still observed 24 h after irradiation for all doses (Figure 4C). Maximum foci numbers were already detected 30 min after irradiation with carbon ions. Foci occupancy was also significantly elevated 24 h after 0.5 and 2 Gy of carbon ion irradiation in Caco-2 cells (Figure 4D).

For carbon ion experiments, we additionally correlated the number of γ-H2AX foci with the number of ion traversals (Table 1). This was calculated by dividing the nuclear area of the cells (PC3 or Caco-2) by the fluence (different for each dose). The higher the number of ions passing the cell nucleus, the higher the number of foci that we counted after carbon ion irradiation. In addition, the (slightly) higher number of ions that pass the cell nucleus for Caco-2 cells compared to PC3 cells correlates with the higher number of foci that were counted in Caco-2 cells compared to PC3 cells 30 min after carbon ion irradiation.

**Table 1:** **Ion traversals per cell nucleus were calculated for PC3 and Caco-2 and compared to the results of γ-H2AX foci 30 min after carbon ion exposure**.

	PC3		Caco-2	
	Number of traversals calculated	Number of foci counted after 30 min	Number of traversals calculated	Number of foci counted after 30 min
0.5 Gy	12.5	0.9	15.9	5.1
1 Gy	25.1	16.7	31.7	23.2
2 Gy	49.9	19.2	63.1	29.8

*The number of traversals was calculated by dividing the nuclear area of the cells (PC3 or Caco-2) by the fluence (different for each dose). Nuclear area for PC3 cells was on average 134.7 μm and for Caco-2 cells 170.5 μm*.

### Cell Cycle Analysis

Radiation-induced cell cycle changes were analyzed by flow cytometry at 24, 48, and 72 h after X- and carbon ion irradiation using PI staining. Representative histograms are shown in Figure 1 for both PC3 and Caco-2 cells.

**Figure 5:**
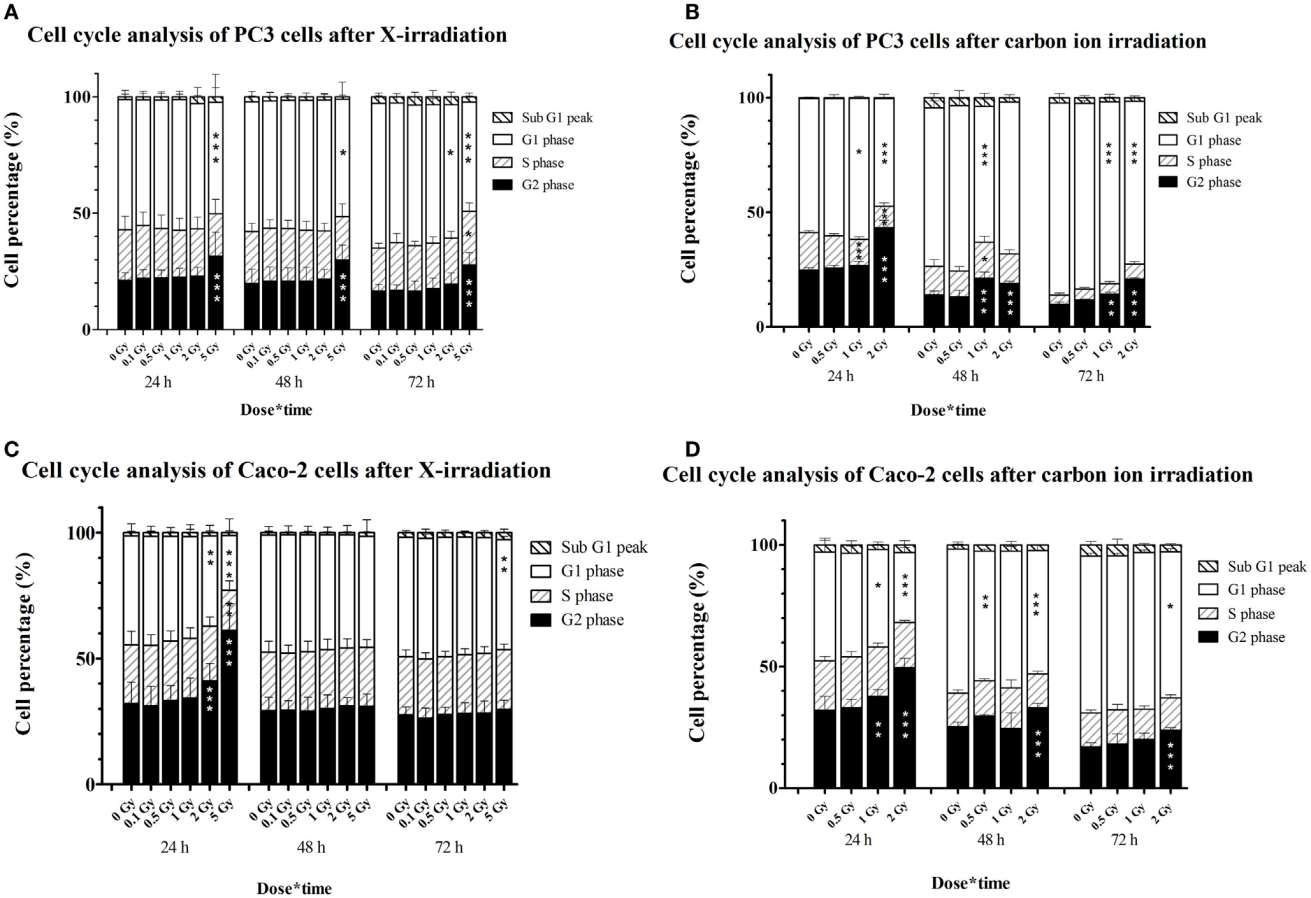
**Cell cycle distribution of irradiated PC3 and Caco-2 cells assessed by PI staining and flow cytometry**. Stacked graphs representing percentages of cells per cell cycle phase in PC3 cells irradiated with X-rays **(A)** or carbon ions **(B)** and in Caco-2 cells irradiated with X-rays **(C)** or carbon ions **(D)**. Bars represent an average of three experiments for X-irradiated samples and one experiment for the carbon ion-irradiated samples. Statistical two-way ANOVA with Bonferroni *post hoc* test was performed in GraphPad Prism with *p < 0.05 (vs. control cells), **p < 0.01, and ***p < 0.001 (vs. control cells).

In PC3 cells, 5 Gy of X-irradiation resulted in an increase of the percentage of cells in G2 phase (~10%) at all time points at the expense of G1 cells (Figure 5A), suggestive of a persistent G2/M arrest. Lower doses of X-rays did not affect the cell cycle of PC3 cells. On the other hand, carbon ion irradiation of PC3 cells resulted in a significant increase of cells in G2/M-phase, 24 h after 2 Gy and 48 and 72 h after both 1 and 2 Gy (Figure 5B). This was combined with a decrease in cells in G1 phase at all time points both at 1 and 2 Gy. After 1 Gy carbon ion irradiation, significant changes in the fraction of S-phase cells were found after 24 and 48 h.

In Caco-2 cells, a dose of 2 and 5 Gy of X-rays increased the number of cells in G2/M-phase, although only transiently (Figure 5C). This was combined with a decrease in the number of cells in G1 for both doses and a decrease in cells in S-phase for 5-Gy X-irradiation. After 48 and 72 h, the G2/M arrest was resolved in Caco-2 cells irradiated with X-rays. However, a small but significant decrease (almost 4%) in G1 phase cells was found at 72 h after 5-Gy X-irradiation. Irradiation of Caco-2 cells with 2 Gy of carbon ions resulted in a persistent G2/M arrest, accompanied by a decrease of cells in G1 phase (Figure 5D). At the earliest time point, this could also be observed after 1 Gy carbon ion irradiation.

## Discussion

From a physical point of view, the rationale for the use of particle irradiation in cancer therapy has been clear for a very long time. Along with the positive patient responses observed in clinical trials using particle therapy, it has been of increasing interest to understand and unravel the underlying biological mechanisms and pathways involved by means of *in vitro* studies. Important differences between both radiation qualities in DNA damage and subsequent cell cycle arrest have been indicated (33), which explain the higher RBE induced by particle radiation. In this study, we investigated changes in DNA damage and repair kinetics of PC3 and Caco-2 cell lines exposed to carbon ion or X-irradiation. In addition, cell cycle stages in both cell lines were analyzed. We observed an increase in γ-H2AX foci number and foci occupancy after X-irradiation with some interesting differences between both cell lines. The initial induction of γ-H2AX was similar for both cell lines although foci occupancy was higher in PC3 cells than in Caco-2 cells after exposure to X-rays. One explanation for this could be the difference in radiosensitivity between both cell lines, as we previously observed (29). Exposure to carbon ions resulted in a higher initial induction of γ-H2AX foci for Caco-2 cells compared to PC3 cells. In samples exposed to X-rays relatively less residual damage after 24 h was observed in Caco-2 cells compared to PC3 cells (mean foci count after 5 Gy was 25 foci after 30 min and 20 foci after 24 h in PC3 cells, and 26 foci after 30 min and 8 foci after 24 h in Caco-2 cells). This lower residual damage observed in Caco-2 cells after X-irradiation can also be linked to a higher surviving fraction of Caco-2 cells compared to PC3 cells as we observed previously (29).

We found no reports on γ-H2AX analysis of irradiated Caco-2 cells and only one for PC3 cells (34). They irradiated confluent PC3 cells with 2 Gy X-rays and visualized γ-H2AX foci after 30 min and 24 h. After 30 min, 10 foci were observed after 2 Gy of X-rays, compared to 5 foci in our PC3 cells. However 24 h after exposure we found a higher residual number of γ-H2AX foci in the PC3 cells (i.e., 7 foci observed by van Oorschot vs. 12 foci observed in our study). One explanation for this could be the different set-up of the experiment; more specifically van Oorschot et al. used a dose rate of 3 Gy/min, whereas we used a dose rate of 0.25 Gy/min. Another explanation could be a difference in the confluence of the irradiated cells, which could synchronize the cells in a certain phase making the cells more or less resistant to the effect of (X-ray) irradiation.

Our data showed that 30 min after exposure to carbon ions, a higher number of foci were induced at a therapeutic dose of 2 Gy compared to X-rays. More specifically, in PC3 cells, we observed five radiation-induced foci after irradiation with 2 Gy of X-rays compared to 19 foci after an equal dose of carbon ions. For Caco-2 cells, the number of radiation-induced foci after 2 Gy of X-rays and carbon ions was 8 and 30, respectively. This is in contrast to a study by Ghosh et al. (15) in which A549 cells were irradiated with γ-rays (1, 2, or 3 Gy) or ^12^C ions (1 Gy, 5.2 MeV/u; LET = 290 keV/μm). They observed that equal doses of both radiation qualities induced similar numbers of foci 15 min after irradiation.

A closer look at the residual foci number (at 24 h) after 2 Gy irradiations shows that less foci are detected in carbon ion-irradiated PC3 samples compared to X-ray samples (i.e., increase of 6 foci after carbon ion irradiation; increase of 12 foci after X-rays;). However, we should note that samples exposed to carbon ions were irradiated in a vertical position, perpendicular to the irradiation beam. Since carbon ion irradiation is expected to induce more complex damage along the ionization tracks, more foci would be present behind one another along the *Z*-axis. This could explain why although less foci are counted in general and less are present after 24 h, the residual damage could still be more complex, which, in turn, explains the persistent G2/M arrest we observed after both 1 and 2 Gy carbon ion irradiation. Additionally, because we analyzed the foci in the same direction as the position of the irradiation beam, it is possible that spots overlapped, causing the foci number to be lower than expected (35). Similar observations were made by a study of Rall et al. in which human blood-derived cells were irradiated with 2 Gy of high-LET irradiation (iron ions, LET = 155 keV/μm). Because of the higher RBE of iron ions, a higher induction of γ-H2AX foci for iron ion-irradiated samples compared to the X-ray irradiated samples was expected, but not observed. The authors hypothesized that the formation of γ-H2AX foci along the beam track has a limited resolution, leading to lower foci numbers (12, 36, 37). In Caco-2 cells, however, we measured lower levels of residual γ-H2AX foci after 24 h in X-irradiated samples compared to carbon ions (i.e., increase of 2 foci after 2 Gy X-rays; increase of 7 foci after 2 Gy carbon ion irradiation). Also here, damage is expected to be more complex and could, therefore, be responsible for the persistent G2/M arrest induced by carbon ions, which was not observed after X-irradiation.

As could be expected, carbon ion irradiation was more potent in inducing cell cycle arrest as compared to equal doses of X-ray irradiation. A persistent G2/M arrest was observed in PC3 cells, already after a dose of 1 Gy of carbon ions. By contrast, only a dose of 5 Gy X-rays was able to induce a persistent cell cycle arrest in PC3 cells (up to 72 h post irradiation). For Caco-2 cells, 2 Gy carbon ion irradiation was capable of inducing a persistent G2/M arrest, whereas after X-radiation Caco-2 cells seemed to escape from the G2/M arrest 48 h after irradiation. These differences indicate the potency of particle radiation to induce more severe damage that can lead to (persistent) cell cycle arrest. In Caco-2 cells, a transient G2/M arrest was observed after X-irradiation; whereas in PC3 cells, this arrest persisted until 72 h after exposure. These different results could be explained by the lower residual DNA damage that we observed after 24 h in Caco-2 cells compared to PC3 cells. Another explanation could be the difference in doubling time between both cell lines, where PC3 cells have a higher doubling time compared to Caco-2 cells.

To our knowledge, no previous studies investigated the effect of particle irradiation on cell cycle progression of Caco-2 cells, while only one study investigated cell cycle changes in PC3 cells after proton irradiation (38). In their study, cells were exposed to 10, 20, or 40 Gy of either photon or proton irradiation. With regard to cell cycle changes, they observed a less pronounced and delayed G2/M arrest after photons compared to proton irradiation. This is consistent with our and previously published results comparing various cell lines irradiated with different beam qualities (25, 39–42). However, most of these studies only focused on cell cycle changes up to 24 h post irradiation. We analyzed as far as 72 h after irradiation and found that, compared to X-rays, a lower equal dose of carbon ions was sufficient to induce a permanent G2/M arrest in PC3 cells. For Caco-2 cells however, a qualitative difference in cell cycle arrest was observed. To this regard, we demonstrated that a lower dose of carbon ion particles was capable of inducing a persistent arrest that was not present after X-rays.

Differences in repair kinetics between X- and carbon ion irradiation, as we observed here, might be an indication of activation of different DNA repair pathways due to differences in the complexity of the DNA damage (37, 43, 44). In this context, it is also important to note that the genetic background of the tumor will influence the effectiveness of radiotherapy. The cell lines we used in this study do not express p53, as described in the literature (45–48) and this lack of p53 expression was confirmed for both our cell lines (data not shown). As mentioned before, p53 is normally activated in response to DNA damage and induces cell cycle arrest. Since p53 can control both G2/M and G1 cell cycle check points (49, 50), our data suggest that, at higher doses of X-rays, p53-independent mechanisms are responsible for the observed G2/M arrest. This may partly explain the radioresistance of both cell lines to X-ray therapy. Previous studies have shown that carbon ion-induced cell killing is independent of the p53 status (7, 51–53). On the other hand, the repair of γ-H2AX foci, which can be observed 24 h after exposure, also indicates that p53-independent repair mechanisms are still active within these cell lines. Importantly, our observation that the threshold for p53-independent cell cycle arrest is reached after exposure to lower doses of carbon ion irradiation, while DNA damage repair is less efficient, suggests that carbon ion radiotherapy could be more appropriate to treat radioresistant tumors with a mutated p53 status.

## Conclusion

In the present study, we investigated the acute cellular responses after carbon ion and X-ray exposure in two p53-defective cancer cell lines. First, our results indicate that a higher amount of initial DNA damage is induced by carbon ion irradiation compared to X-irradiation, even when lower doses are used. In addition, repair kinetics of γ-H2AX foci of Caco-2 cells showed relatively more residual DNA damage at 24 h after carbon ion irradiation compared to X-irradiation. Second, cell cycle progression assays demonstrated a persistent cell cycle arrest of PC3 cells, which was induced by lower equal doses of carbon ion compared to X-irradiation. In Caco-2 cells, a persistent arrest was induced by carbon ions but not by X-irradiation. Further research is needed to better understand how different radiation qualities influence acute cellular responses, which are in part responsible for the increased biological effectiveness of particle beam irradiation.

## Author Contributions

AS performed experiments both at SCK•CEN and GANIL. KK performed experiments at SCK•CEN. MM designed the experimental set-up and performed experiments at GANIL. RQ helped with analysis and interpretation of obtained data. MV helped with microscopical analysis of obtained data. ES helped with experiments performed at GANIL and SCK•CEN. KT helped with experiments performed at GANIL and SCK•CEN. VG contributed to the design of the work. SB contributed to the design of the work, as well as with interpretation of obtained data. All co-authors critically reviewed and approved the final version to be submitted to this Journal.

## Conflict of Interest Statement

The authors declare that the research was conducted in the absence of any commercial or financial relationships that could be construed as a potential conflict of interest.
